# Effect of Orento, a Traditional Japanese Medicine, on IL-6, IL-8 Secretion, Type 1 Collagen Production and Alkaline Phosphatase Secretion in the Human Osteosarcoma Cell Line Saos-2

**DOI:** 10.3390/medicines7100061

**Published:** 2020-09-25

**Authors:** Hourei Oh, Kazuya Masuno, Nobutaka Okusa, Yoshimasa Makita, Shin-ichi Fujiwara, Yasuhiro Imamura

**Affiliations:** 1 Department of Innovation in Dental Education, Osaka Dental University, Osaka 573-1121, Japan; masuno@cc.osaka-dent.ac.jp; 2 Department of Forensic Dentistry, Osaka Dental University, Osaka 573-1121, Japan; okusa-n@cc.osaka-dent.ac.jp; 3 Department of Chemistry, Osaka Dental University, Osaka 573-1121, Japan; makita@cc.osaka-dent.ac.jp (Y.M.); fujiwara@cc.osaka-dent.ac.jp (S.-i.F.); 4 Department of Dental Pharmacology, Matsumoto Dental University, Nagano 399-0781, Japan; yasuhiro.imamura@mdu.ac.jp

**Keywords:** orento, traditional Japanese medicine, anti-inflammatory, periodontal disease, ALP, collagen

## Abstract

**Background:** Orento, a traditional Japanese medicine, is known as Kampo medicine in Japan. We investigated the possible efficacy of Kampo medicine for periodontal disease. In this study, we examined the in vitro effects of orento on the proliferation of the inflammatory cytokines interleukin (IL)-6 and IL-8, the production of type 1 collagen, and the secretion of alkaline phosphatase (ALP) in the human osteosarcoma cell line Saos-2 (Saos-2 cells). **Methods:** The proliferation of Saos-2 cells was assessed by MTT assay. IL-6 and IL-8 levels, type 1 collagen production and ALP secretion were evaluated using enzyme-linked immunosorbent assay and ALP assays. Saos-2 cells were treated with or without 0.1, 1, 10, 100 and 1000 μg/mL of orento for 24 h. **Results:** Orento (10 μg/mL) significantly induced the proliferation of Saos-2 cells. At this concentration, orento suppressed IL-6 and IL-8 and enhanced type 1 collagen production and ALP secretion. **Conclusions:** These results indicate that orento controls the IL-6 and IL-8 secretion and cellular metabolism of osteoblasts, resulting in the secretion of early bone-related biomarkers.

## 1. Introduction

Kampo medicines, or traditional Japanese medicine, originated from ancient Chinese medicine. Kampo medicines have characteristics such as abdominal diagnosis for therapeutic indications and useful formulas with smaller amounts of herbs, compared with Chinese medicine [1,2,3]. In 1967, four Kampo medicines were covered by National Health Insurance in Japan. At present, 148 Kampo medicines are approved as prescription drugs. In general, Kampo medicines are extracts of herbal formulas and prepared as described in the classical Kampo literature.

Orento is a type of Kampo medicine composed of seven crude drugs. These include *Coptis* rhizome, processed ginger, cinnamon bark, *Pinellia* tuber, ginseng, *Glycyrrhiza* and jujube. Orento is mainly used to treat symptoms such as feelings of stagnation and disturbance of stomach and loss of appetite [4]. In clinical practice, orento is indicated for the treatment of acute gastritis, hangover, stomatitis and periodontitis [5].

We previously established a human gingival fibroblast culture system stimulated with the lipopolysaccharide (LPS), an endotoxin, of the periodontitis-associated bacteria *Porphyromonas gingivalis* [6]. Using this system as an in vitro cellular model for periodontal disease, we demonstrated the efficacy of a variety of herbal medicines, including shosaikoto, orengedokuto, hangeshashinto, hochuekkito, hainosankyuto and orento, for the treatment of periodontitis [7]. The previous study showed that orento suppresses *Porphyromonas gingivalis* LPS-induced extracellular signal-regulated kinase phosphorylation and the consequent cytoplasmic phospholipase A2 phosphorylation that results in the production of prostaglandin (PG) E2 by human gingival fibroblasts. Thus, one of the mechanisms of the anti-inflammatory action of orento was elucidated from this study [8].

Pathologically, in periodontal diseases, periodontal tissues such as the gingiva and the alveolar bone are affected owing to inflammation. Cells of infected tissues secrete inflammatory cytokines by bacterial components of LPS and peptidoglycan. IL-6, which is a representative of inflammatory cytokines, activates osteoclasts and IL-8 acts as a chemotactic factor for neutrophils and induces an inflammatory response [6]. Therefore, we were interested in determining the influence of orento on osteoblasts. The human osteosarcoma cell line Saos-2 has high alkaline phosphatase (ALP) activity and was selected as the osteoblast differentiation marker [9]. To date, orento has not been studied using Saos-2 cells.

In this study, we investigated the in vitro effect of orento on IL-6 and IL-8 secretion, type 1 collagen production and ALP secretion in Saos-2 cells.

## 2. Materials and Methods

### 2.1. Cell Cultures

Saos-2 cells (RIKEN BRC Cell Bank) were cultured in Dulbecco’s modified Eagle’s medium (DMEM, Nissui Pharmaceutical Co., Ltd., Tokyo, Japan) with 10% fetal bovine serum (FBS), 100 units/mL penicillin G and 100 µg/mL streptomycin at 37 °C in a 5% CO_2_ and 95% air humidified incubator [9].

### 2.2. Reagents

The following materials and antibodies were purchased: orento (Tsumura, Tokyo, Japan), anti-interleukin (IL)-6 and biotinylated anti-IL-6 antibodies (eBioscience, Inc., San Diego, CA, USA), anti-IL-8 and biotinylated anti-IL-8 antibodies (R&D Systems, Inc., Minneapolis, MN, USA), biotinylated anti-collagen type I antibody (Rockland Immunochemicals, Inc., Limerick, PA, USA) and 3-(4,5-dimethylthiazol-2-yl)-2,5-diphenyl tetrazolium bromide (MTT) (Sigma-Aldrich, St. Louis, MO, USA). *Aggregatibacter actinomycetemcomitans* (*A. a.*) LPS was a kind gift from Dr. H. Senpuku (National Institutes of Health, Kawasaki, Japan) [10,11,12]. No LPS contamination was confirmed in purchased orento. Orento (10 mg/mL) stock solution was prepared by the following procedure: One-hundred milligrams of orento was added to 10 mL of distilled water and stirred for 9 h at room temperature. The crude solution was centrifuged at 5000 rpm for 5 min. The supernatant liquid was filtered by syringe filter (0.2 μm, Sartorius Co., Ltd., Göttingen, Germany).

### 2.3. MTT Assay

Saos-2 cells (1 × 10^4^) were cultured with orento at concentrations of 0.1, 1, 10, 100 and 1000 µg/mL in DMEM containing 10% FBS for 24 h, and the culture medium was then removed. The remainder of the procedures were performed as described in a previous study [13].

### 2.4. Enzyme-Linked Immunosorbent Assay (ELISA)

For evaluation of cytokine production, the Saos-2 cells (1 × 10^4^) were cultured with mixtures of A. a. LPS (100 ng/mL) and orento (10 µg/mL) for 24 h. Following this, the culture media were collected, and the cytokine levels were measured using the anti-IL-6 (1 μg/mL) and biotinylated anti-IL-6 (0.6 µg/mL) or anti-IL-8 (2.5 µg/mL) and biotinylated anti-IL-8 (0.2 µg/mL) antibodies. To evaluate collagen production, Saos-2 cells (1 × 10^4^) were cultured in DMEM containing 1% FBS with 10 µg/mL of orento for 24 h. The levels of type 1 collagen in the media were measured using the biotinylated anti-collagen type I antibody (0.2 µg/mL). The instructions in the user manual of the CytoSet kits (Thermo Fisher Scientific, Waltham, MA, USA) were followed to perform the ELISA tests [14]). The cells prepared for the evaluation of collagen production were lysed with 0.5% Triton X-100 and the protein concentration in the cell lysates was measured using a bicinchoninic acid (BCA) protein assay kit (Thermo Fisher Scientific, Waltham, MA, USA). Collagen production was normalized to the protein content of the cell lysates.

### 2.5. ALP Activity

Saos-2 cells (1 × 10^4^) were cultured with orento (10 µg/mL) for 24 h. The cells were lysed with 0.05% Triton X-100 and the ALP activity of the cell lysates was measured using a LabAssay ALP kit (Wako). The amount of protein in the cell lysates was also measured using a BCA protein assay kit. ALP activity was normalized to the protein content of the cell lysates [9].

### 2.6. Statistical Analysis

Quantitative data were statistically analyzed using either one-way analysis of variance followed by Tukey’s test (ELISA of IL-8 production) or Student’s *t*-test (ALP assay and ELISA of collagen production) using the StatMate software (ATMS). Differences were considered significant at a value of *p* < 0.05.

## 3. Results

**Figure 1:**
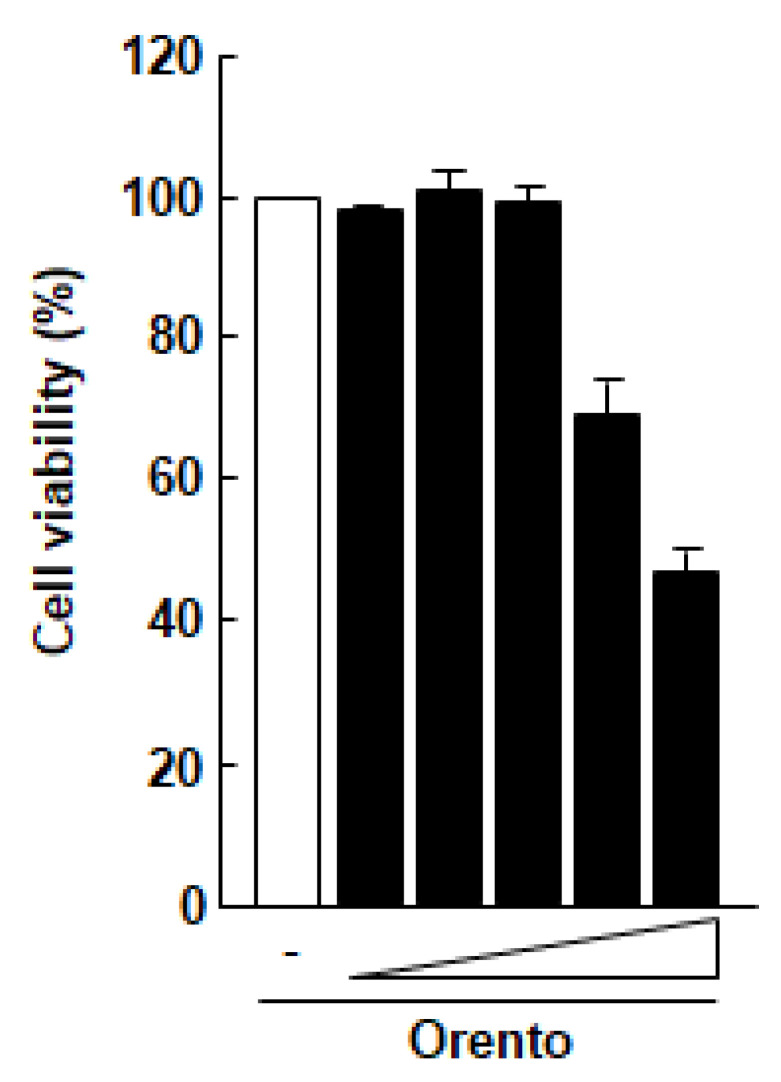
Effect of orento on the proliferation of Saos-2 cells.

The effect of orento on the viability of Saos-2 cells was examined using a MTT assay (Figure 1). At doses of up to 10 μg/mL, orento showed no marked cytotoxicity against Saos-2 cells. These findings suggest that orento at this level may be cytotoxic against Saos-2 cells. Therefore, the conditions of this study were narrowed down to a range within which the orento did not exhibit cytotoxicity against Saos-2 cells, and the following experiments were carried out using orento at a dose of 10 μg/mL. The substrate had no effect on cell proliferation (data not shown).

**Figure 2:**
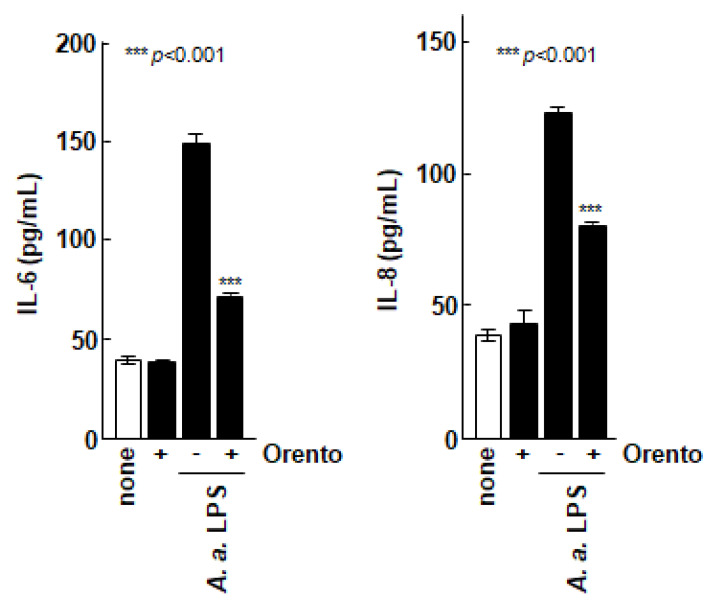
Effect of orento on inflammatory cytolysis of Saos-2 cells. *** is *p* < 0.001.

We then evaluated the effect of orento on the secretion of inflammatory cytokines by Saos-2 cells as shown in Figure 2. The production of IL-6 and IL-8 by Saos-2 cells was markedly promoted when the Saos-2 cells were stimulated with LPS from the periodontal pathogenic bacterium *A. a.*, which is known to have a strong inflammation-inducing effect. In contrast, the production of ILs was markedly suppressed when the Saos-2 cells were stimulated with LPS in the presence of orento.

**Figure 3:**
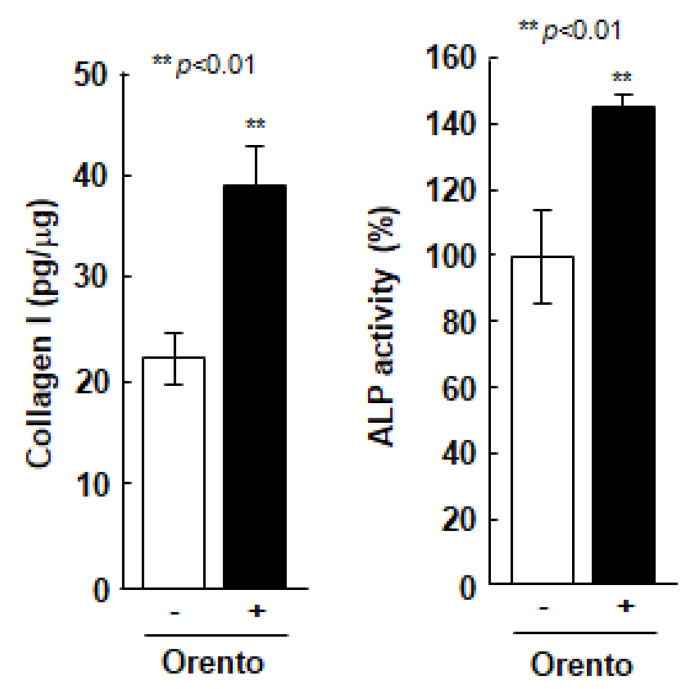
Collagen type 1 production in Saos-2 cells treated with orento and ALP secretion in Saos-2 cells treated with orento. ** is *p* < 0.01.

The effect of orento on the ability of Saos-2 cells to produce type 1 collagen, was directly linked to the regenerative capacity of periodontal tissues (Figure 3). The level of orento, that promoted cell proliferation compared to the controls during the evaluation of cell viability, also enhanced the production of type 1 collagen in Saos-2 cells by approximately 1.4-fold and enhanced ALP secretion (Figure 3) in Saos-2 cells compared to the Saos-2 cells without orento treatment. Furthermore, 10 μg/mL of orento promoted the production of type 1 collagen and secretion of ALP, both of which are highly related to bone formation.

Saos-2 cells seeded at a volume of 3.1 × 10^4^/cm^2^ were exposed to media containing 0.1, 1, 10, 100 and 1000 μg/mL for 24 h. All data were compared with the data obtained for cells treated with control medium without orento.

To investigate the inhibitory effects of orento on IL-6 and IL-8 secretion by control Saos-2 cells or Saos-2 cells stimulated with *Aggregatibacter actinomycetemcomitans* lipopolysaccharide (LPS). Saos-2 cells were exposed to media containing 10 μg/mL of orento for 24 h. IL-6 and IL-8 concentrations in the supernatants were measured using enzyme-linked immunosorbent assay. Data are presented as mean ± standard deviation (n = 5). *** *p* < 0.001, analyzed using analysis of variance with a Tukey’s test. Statistical differences among orento (+/−) exposures upon LPS administration are shown in figure.

To investigate the collagen type 1 production in Saos-2 cells treated with orento, Saos-2 cells were seeded at a volume of 3.1 × 10^4^/cm^2^ and were exposed to media containing 10 μg/mL of orento for 24 h. Data are presented as mean ± standard deviation (n = 3). ALP secretion in Saos-2 cells treated with orento. Saos-2 cells were seeded at 3.1 × 10^4^/cm^2^ and were exposed to media containing 10 μg/mL of orento for 24 h. Data are presented as mean ± standard deviation (n = 3). ** *p* < 0.01, analyzed using Student’s *t*-test (vs. none).

## 4. Discussion

Orento is a drug whose source is the Shang Hang Lun [4]. In dental practice in Japan, orento is used for patients with stomatitis and periodontal disease. Furthermore, we previously used an in vitro periodontal disease model to characterize the anti-inflammatory effects of orento [7]. However, the current study presents challenges in elucidating the exact mechanism of the effect of orento on Saos-2 cells, as is typically done in Western medicine, given that traditional Japanese medicines are often a cocktail of a variety of crude ingredients [5].

Orento contains seven crude drugs (Table 1). In Japanese herbal prescriptions, all constituent herbal medicines do not have the same importance, but some of them support the important core herbs and their actions. Traditional medicines are composed of herbal ingredients that allow the central herbal ingredient to exert its full effect. The central herbal ingredient is commonly referred to as the “emperor herb,” and the crude drugs that assist and strengthen the actions of the “emperor herb” are called “minister herbs.” Additionally, there is an auxiliary role of an assistant herb to the emperor and minister herbs and this assistant is known as the “harmonizer herb.” In orento, the platycodon root [5] Coptis rhizome is the emperor herb and contains the alkaloid berberine (5–10%) [1]. Therefore, we have focused our discussion on berberine in the emperor herb *Coptis* rhizome.

Many plants are known to have a wide range of biologic and medicinal properties. The secondary metabolites of alkaloids, which are naturally occurring organic compounds containing nitrogen atoms, have various pharmacological properties. Therefore, crude drugs have hitherto been used to prevent and treat systemic diseases as well as oral diseases such as stomatitis. A Chinese medicine that is an extract of yellow lotus is known to contain a large amount of berberine, which is an isoquinoline alkaloid [15].

The anti-inflammatory effects of berberine have been previously reported [16], and its active component has also been isolated [17]. Furthermore, it has been suggested that berberine exerts its anti-inflammatory effect by suppressing mitogen-activated protein kinase (MAPK) signaling and production of reactive oxygen species [18]. It has been shown that berberine suppresses LPS-induced inflammatory cytokine production in macrophages [19]. Animal models of diabetic nephropathy suggest that treatment with berberine inactivates nuclear factor-kappa B and suppresses kidney inflammation [20]. Recently, in order to elucidate the mechanism by which NF-E2-related factor 2 (Nrf2) suppresses inflammation, we analyzed Nrf2 target genes in macrophages and revealed that Nrf2 suppresses inflammation by inhibiting the expression of IL-6 and IL-1β genes. Therefore, it can be concluded that substances that can suppress the production of IL-6 are candidates for anti-inflammatory drugs [21]. Based on these reports, it is believed that the suppression of IL-6 inflammatory cytokines in this study was due to orento and the berberine contained in the oriental drug Oren, which supports the results of this study.

However, IL-8 is an inflammation-related mediator that plays a key role in neutrophil recruitment and degranulation [22]. In periodontal disease, IL-8 induces an inflammatory reaction and cell damage through a complex cytokine network and tissue destruction process [23].

Studies have shown that berberine suppresses the activity of matrix metalloproteinases (MMP)-2 and pro-MMP-2 in human gingival fibroblasts that are stimulated by LPS derived from periodontal disease–causing bacteria [12,24]. For example, the macrolide antibacterial drug erythromycin is said to increase apoptosis by human neutrophils, decrease IL-8 secretion and have an anti-inflammatory effect [25]. Based on the data from these previous reports, our study suggests that orento may have an anti-inflammatory effect because it suppresses IL-8.

Another study has reported that when recurrent aphtha was treated by gargling a liquid formulation containing berberine, a significant improvement was noted compared with gargling a formulation without berberine [26]. In Japan, the use of a berberine-containing gargling solution for oral mucositis caused by radiotherapy has shown good therapeutic results [27].

Furthermore, recent studies have suggested that berberine is useful for inducing bone regeneration [28]. Berberine suppresses the receptor activator of NF-κB ligand (RANKL) gene expression in osteoblasts through the NF-κB pathway, nuclear factor of activator T cell pathway and phosphoinositide 3 kinase/Akt pathway and enhances osteoprotegerin (OPG) gene expression. As a result, it has been suggested that berberine is useful for the destruction of inflammatory tissues by suppressing the differentiation of osteoclasts [29,30]. Furthermore, when preosteoblasts were stimulated with berberine, the expression of bone differentiation markers OPG, osteocalcin (OCN) and Runt-related transcription factor 2 (Runx2) was enhanced through the activation of p38MAPK, thus promoting calcification. Therefore, berberine may be useful for bone regeneration [31]. It has been reported that berberine stimulates bone marrow mesenchymal stem cells to enhance the expression of OPG, OCN and Runx2 via the Wnt/β-catenin pathway, suggesting that berberine induces bone differentiation [32]. Additionally, in situ staining tests have revealed that berberine enhanced ALP enzyme activity [33]. In our current study, orento induced the expression of type 1 collagen and ALP secretion in osteoblast precursor cells. Osteoblasts gradually differentiate into Runx2, type 1 collagen, ALP, osteopontin and bone sialoprotein. Osteoblasts express bone matrix proteins such as osteocalcin. Therefore, the possibility of bone formation induced by orento cannot be ruled out.

The main pathogenic factors in periodontal disease such as periodontopathic bacteria, fimbriae, proteases and endotoxins, act on monocytes, macrophages, gingival fibroblasts, IL-1β, IL-6, IL-8, PGE2, tumor necrosis factor-α, chemokine (CC-motif) ligand 5 and MMPs to induce inflammatory reactions and cell damage through a complex cytokine network and eventually alveolar bone destruction progresses [23]. Combined with our previous studies [7,8], our current study suggests that orento may possess anti-inflammatory effects in the context of periodontitis and also has osteoblast activation effects.

In summary, we demonstrated the effect of orento in vitro on IL-6, IL-8 secretion, type 1 collagen production and ALP secretion in the human osteosarcoma cell line Saos-2.

## Figures and Tables

**Table 1:** Crude drug components of orento.

Herb Levels	Crude Drug	Ratio
Emperor herb	*Coptis* rhizome	3
Minister herbs	Processed ginger	6
	Cinnamon bark	3
	*Pinellia* tuber	3
Assistant herb	Ginseng	3
	*Glycyrrhiza glabra*	3
Harmonizer herb	Jujube	3
